# Tuberculosis in Relation to Animal Industry and Public Health1Read before the Ontario Veterinary Medical Association, Toronto, December 21, 1894.

**Published:** 1895-01

**Authors:** Anderson Crowforth

**Affiliations:** Lockport, N. Y.


### TUBERCULOSIS IN RELATION TO ANIMAL INDUSTRY AND PUBLIC HEALTH.[1]

### Its Prevalence and Relative Importance.

BY ANDERSON CROWFORTH, V.S.

Tuberculosis is so extensively prevalent and proves such a veritable scourge throughout the civilized world that no disease is so deserving of close and accurate study or of the enforcement of effective measures for its suppression. Cholera, yellow-fever, and smallpox, which occasionally invade our territory, creating universal terror and dismay, claim but few victims as compared with this ever-present, universally devastating plague. These other plagues are quick, severe, and fatal, it is true, but for this very reason they can be promptly recognized and checked, and even stamped out; whereas, tuberculosis is equivocal and underhanded in its method, slow and uncertain in its progress, and on this account escapes recognition and proves by far the most deadly of any single disease attacking the human family.

[1] Read before the Ontario Veterinary Medical Association, Toronto, December 21, 1894.

The average ratio of deaths from tuberculosis to the total mortality is 14 per cent., or one death in every eight; while under special conditions it rises to one in three, as in the Marquesas Islands, or even one in two, as on some of our Indian reservations. Tuberculosis may be classed with "the pestilence that walketh in the darkness," while the other three diseases named are like "the destructions that wasteth at noonday." But the deaths from tuberculosis being constant and uniform, people accept them as inevitable and fold their idle hands with true Mohammedan fatalism, instead of boldly exposing the hidden death-trap and cutting short its destructive work.

If the 5490 deaths from tuberculosis which occur every year in the city of New York could be brought together in an epidemic lasting but one week, no smallpox, cholera, nor yellow-fever scare would approach the panic which would thus be created: for did all these diseases together create such mortality in that city? Nay, if we take the whole civilized world and compare with the tuberculosis mortality all the accumulated deaths from war, famine, plague, cholera, yellow-fever, and diphtheria and smallpox, we find that the latter are comparatively very insignificant. Yet tuberculosis, like every germ disease, is absolutely preventable, and is allowed to continue its career of death only because of reprehensible ignorance and criminal indifference.

Its prevalence in the lower animals. Few, if any, diseases maintain a sway over a more numerous genera of animals than tuberculosis. Among the domesticated animals, cattle are the most susceptible, but chickens, guinea-pigs, rabbits, swine, and goats become victims almost, if not quite, as easily. Some have thought that dogs, cats, sheep, and horses are exempt, but when inoculated with tuberculous material these all contract the affection readily enough. The fact that they do not contract it in such numbers in the usual way is probably due in part to the greater amount of outdoor life which they enjoy, also to the fact that they have more exercise, which secures for them a better developed and higher conditioned muscular system, and a full stock of constitutional vigor. After making all these allowances, however, it must be granted that these four classes of animals enjoy a native intolerance to this disease to which the first six classes mentioned are comparative strangers.

Among the less domesticated animals which contract tuberculosis may be named caged apes, lions, kangaroos, deer, elk, gazelle, antelope, birds, and, in addition, the rats, mice, and other vermin of our houses and barns. All must, therefore, be considered as possible bearers and disseminators of the infection, and no such animal (indeed, scarcely any animal) can be left out of account in any systematic attempt to root out the disease. Some, however, are justly held to contribute more than others to the maintenance of the affection, and in this sense, in addition to man himself, we must consider as pre-eminently bearers of this disease cattle, fowls, and pigs.

Accurate statistics are wanting to give the ratio of tuberculous animals in our herds, as we have no systematic professional inspection of live animals and of those killed for human food.

TUBERCULOSIS CONTAGIOSUS. In the middle ages tuberculosis in animals was recognized as contagious, and laws were made against the use of affected carcasses as human food, which remain in force in Italy and Spain to the present day. In the sixteenth century the disease was confounded with syphilis, and at the end of the eighteenth century with glanders; blunders which, however unintentional, show the strong conviction that the malady was contagious. The propagation by contagion in herd was recorded in Germany by Ruhling (1774) and Krunitz (1767), and more recently by Spinola, Zannger, and others. In France the same is claimed by Fromage, Huzard, Taposse, Dupont, and Cruzel.

It must be allowed, however, that in the first half of the present century the manifest tendency of the disease to run in families, and to develop under special unwholesome conditions of life, served to weaken the belief in contagion, and in central and western Europe such belief had become practically extinct among medical men, when their attention was called to the subject by the successful inoculations of tuberculosis on rabbits and guinea-pigs by Villemin in 1865. The subject was taken up on all sides by incredulous experimenters, and for a time a keen polemic warfare raged; but slowly the stern logic of constantly accumulating and unanswerable facts compelled all candid observers to accept the doctrine of contagion.

THE GERM BACILLUS TUBERCULOSIS. An even fuller demonstration came in 1882, when Robert Koch, of Berlin, demonstrated the existence of the tubercle bacillus, and showed that the disease could be produced with equal certainty by inoculating with the substance of a tubercle from the lung of an ox or with a pure culture of the germ grown on peptonized gelatin, apart from the living body.

Before publishing the discovery Koch demonstrated the presence of the bacillus in the expectoration of tubercle of over 100 cases of consumption, and had successfully inoculated 472 subjects—guinea-pigs, rabbits, mice, rats, and cats, besides dogs, pigeons, and chickens. The following are some of the characteristics of the germ:

FORM. A delicate rod, rounded ends, 1.5 to 3.5 micromillimetres in length (or about $\frac{1}{2500}$ of an inch). It occurs singly or in pairs, or chains of three or four connected end to end. When cultivated on blood-serum the groups tend to form elongated, rope-like colonies, having a wavy or serpentine outline.

STAINING. It is characteristic of this bacillus that it absorbs coloring matter very slowly, but, once stained, retains its color with great tenacity.

This enables the microscopist to distinguish this amid a mass of other microbes. The opaque particle in the sputum or a section of tubercle is stained by prolonged exposure to a warm alkaline solution of aniline pigment; it is then bleached by a solution of nitric acid (1:3), and washed and slightly stained with a color which will contrast with the first; finally it is washed, mounted, and examined under the microscope. The rod-like bacillus tuberculosis appears stained with the first color, while the other bacteria, if any, are stained with the second.

BIOLOGY, LIFE-HISTORY. The bacillus tuberculosis lives mainly as a parasite in the animal body, but may be cultivated on the ordinary culture media containing 5 per cent. of glycerin, and makes the best growth at 100° to 102° F. A temperature of 158° F. for ten minutes is fatal to it (Yersin).

Unlike many bacilli, this shows no spontaneous movement at any stage of its growth. Its development is slow in any medium, the earliest signs of growth being visible only after ten or fourteen days.

VITALITY. As it has great power of resistance to the entrance of coloring fluids, so this germ can hold its own for a length of time against destructive agencies. It retains its vitality and infecting power for nine to ten months in dried expectoration (Koch, Schill, Fischer, De Thoma).

Tuberculous cow's lung, dried and pulverized, is virulent after forty-three days (Schill, Fischer), or 102 days (Cadeac, Malet). The bacillus is not destroyed by gastric juice (Baumgarten, Fischer, Falk). In sputum it perishes in twenty hours in a 3

per cent. solution of carbolic acid, in a saturated aqueous solution of salicylic acid, or in saturated aniline water; in five minutes by iodoform ether; in ten minutes in sulphuric ether or mercuric chloride 1 : 1000; in three hours in thymol. It dies in a few hours in direct sunlight, and in five to seven days in diffused daylight. In an ordinary room it gradually weakens, but remains virulent for at least two and one-half months.

INDESTRUCTIBILITY OF THE GERM. *Drying and heating.* The substance of a tubercle, like expectoration, may be dried up and reduced to powder without lessening its virulence, provided too much heat has not been employed in the drying.

Although an absolute heat of 158° F. for fifteen minutes is fatal to the germ, yet in virulent masses, as in meat, it is difficult to determine the actual temperature at all points, and tests are, therefore, often misleading. The interior of broiled steak has been found infected after subjecting it to a temperature of 163° to 176° F. Tuberculous matter heated in sealed tubes to 212° F. has been tried, and even then some germs would exceptionally escape. Some claim that no germs survive the boiling temperature for more than half an hour. Others claim that 162° F., which coagulates albumen, sterilized completely if kept up for a sufficient length of time. If in cooking meat the blood oozes out, the temperature has not been high enough to insure the death of the bacillus. There is a risk of tuberculosis in eating rare steak.

In milk, as in meat, heat is effective at 162° F.; the contained albumin is coagulated, the liquid acquires a boiled flavor, a tendency to constipate, and a diminution of its digestibility and nutritious qualities. In sterilizing this liquid, therefore, for infants and invalids it is better to keep the temperature at 158° Fahr. for a longer period, say, half to one hour continuously.

Drying of the tuberculous matter indoors or in the shade, and apart from the above temperature, and its inhalation as fine dust, are the most common causes of tuberculosis. In one store with a tuberculous clerk the dust raised in sweeping out the store infected clerk after clerk, and a similar rising of the virus in dust is a cause of infection in dwelling-houses, stores, barns, barnyards, and railroad cars. It is now allowed that the infecting matter dried on the handkerchief and shaken out in the air is one of the most prolific sources of infection.

So in animals. A manger smeared with the discharge of a diseased animal retains the virus in an active state and infects the next susceptible animal fed from it. Hence, long before the day of Koch and his discovery of the bacillus, the infection of animal after animal which occupied the same stall in succession had convinced observant owners and veterinarians of the contagion in tubercle.

The bacillus will survive in water and moist earth for an indefinite time at all ordinary temperatures. The germ survives a freezing temperature, and putrefaction is not fatal to it. Heavy salting of meats has been thought to be fatal to the germ in one month. In salting, however, the meat is impregnated unequally in different parts of the same mass, and therefore, as in the case of a temporary heating, this cannot be relied on as a safe measure of disinfection. The comparative indestructibility of the germ under any of these conditions shows that the slow absorption of pigment by the bacillus is significant of an equally tardy penetration by other hurtful agents, and of a corresponding power of resistance to ordinary disinfectants.

ACCESSORY CAUSES OF TUBERCULOSIS. While all must recognize that one essential cause of tuberculosis is the bacillus, yet it is wrong to ignore the fact that many conditions of the animal system and its surroundings contribute to the propagation of the disease or retard its progress.

While none such accessory causes can generate tuberculosis in the absence of the bacillus, yet when that diseased seed is present these conditions often serve to sway the balance toward its advancement and diffusion, or its restriction and suppression. A suitable soil and favorable climate are not less essential for the vigorous growth of the microscopic vegetable microbe than for the Florida moss or the palm. Among the most efficient accessory causes may be named:

1. *Hereditary predisposition.* Consumption runs in families, and it has long been supposed that this was essentially a family —that is, an hereditary—disease. But, as a matter of fact, it is extremely rare to find the offspring tuberculous at birth or before. In many thousands of pregnant tuberculous cows killed in the slaughter-houses of Europe not more than ten tuberculous calves have been found. To the same effect is the fact that in calves under a year old, born of cows 6 per cent. and upward of which prove tuberculous, the ratio of tuberculosis is often below one per thousand. In Saxony, with the ratio of tuberculous cattle 16.5 per cent., that of tuberculous calves was only two per thousand. At Lyons, out of 400,000 calves slaughtered, only five were found tuberculous, and at Munich, out of 400,000 but two tuberculous. Again, in such calves the tubercle was found in most cases in the bowels and adjacent glands, suggesting infection after birth through the milk rather than in the liver and lungs, which would have been its natural seat if conveyed before birth through the blood. Not a few of the obstinate and fatal bowel diseases of sucking children and calves are in reality tuberculosis of the bowels induced by the infected milk. What runs in a family, then, is rarely hereditary disease. It is, in the great majority of cases, only a hereditary susceptibility to the disease—tuberculosis; but it is none the less a fearful legacy, being often so potent that the disease attacks certain families as a matter of course, while other families can count on a practical immunity. In the human family this is notorious. In cattle it is no less remarkable.

2. *Close buildings.* Lack of ventilation renders the air impure by repeated breathing. It is so favorable to the propagation of tuberculosis that it has been looked upon as the sole cause (Macormac). Though now certain that this cannot produce tuberculosis at all in the absence of the germ, yet it is such a potent accessory cause when the bacillus also is present that its importance cannot be too highly appreciated.

For the mild cases of tuberculosis in man, life in the open air, day and night, in a genial climate, with pure air, affords one of the best grounds for the hope of recovery or mitigation.

3. *Dark stables.* Dark stables are usually dirty and ill-ventilated, and as such, lower the general health of the inmates, which are then strongly predisposed to tuberculosis. The darkness, however, acts indirectly in depriving the tissues of the body of their due supply of oxygen. The formation of green pigment (chlorophyll) in plants and of red pigment in the blood globules is due to the action of light. In darkness both disappear. But the red globules of the blood are bearers of oxygen to all parts of the body, and if these globules are deficient the whole body is denied its due aeration. The final result is as if the air contained little oxygen; in other words, as in the case of a closed building without any means of renewing the air.

4. *Insufficient or unwholesome food.* Overtaxing, lack of food, and indigestible or innutritious food agree in producing practical starvation and weakness with increased susceptibility to tuberculosis. Hence this disease is the scourge of the half-starved poor and no less of the rich, who abuse their digestive organs and court chronic dyspepsia. So, too, in our dairy herds, the stimulating ration for milk, the warm drinking water, and warm atmosphere, together with the enforced rest in the stalls for months at a time, and the clean, careful milking, soliciting the gland to act to its extreme capacity, all tend to a lowering of the general health and an increased susceptibility. This sufficiently illustrates how the cow, which has been made a milking machine, and which to this end must produce a calf every year, becomes dangerously susceptible to any tubercle bacillus to which it may be exposed. We have developed most valuable qualities at the expense of hardihood, and we must take the consequences. The argument is not that we should part with acquired powers which give the animal its high value, but that we should recognize the attendant dangers and rigorously exclude the tubercle germ.

5. *Breeding too young.* Breeding of immature animals is a most fruitful accessory cause of tuberculosis, as the demand made upon the system for further growth, for the nourishment of the unborn offspring, and later for the nursing of the calf, or for the dairy yield, undermines the strength and vigor. In different families of that marvellous dairy cow, the Jersey, this has been carried to such an extent that discredit has been thrown upon the whole breed as one especially prone to tuberculosis.

6. *Inbreeding—High breeding.* Inbred families of cattle are proverbially subject to tuberculosis. This is due partly to a resulting constitutional weakness, which often shows itself in an increasing indisposition to breed too near relatives, though still fertile with strangers. More frequently it depends on the intensifying and fixing of personal and family characters. The susceptibility to the germ being equally strong in both parents, becomes intensifying in their common offspring, just as the beef or dairy characteristics are improved. Indeed, the qualities which make an animal valuable for the butcher or milkman are exactly such as favor tuberculosis.

The germ of this disease lives by preference in the lymphatic system, either in the lymphatic glands or in the loose connective tissue forming the lymphatic networks leading to these glands. Now the breeds which are pre-eminent for early maturity and rapid fattening or for a high yield of milk are remarkable for their excess of connective tissue, as shown by the delicate mellow skin, and in the case of the Channel Island cattle by the unusually large lymphatic glands. This helps to explain why certain families of beef-making cattle have been virtually ruined by tuberculosis.

Yet it would be equally wrong to abandon the improvement of our beef and our milking breeds. The improvement already attained is essential to successful competition in the market, and prospective improvement will be no less essential in the future. The true and only real remedy is the extinction and exclusion of the bacillus of tubercle.

7. *Ill health.* All acute and chronic diseases leave the system weak and with less power of resistance to other diseases. Above all, we must fear long-standing diseases, which produce emaciation and weakness; fevers, which interfere permanently with the blood-forming process; diseases of the digestive organs, which hinder the requisite preparation and absorption of nutritive elements, and diseases of the lungs, which form a raw or weakened surface on which the bacillus can grow without hindrance. Again, the germ lives best in a slightly alkaline or neutral medium and is weakened by acid contents of the stomach during vigorous digestion. But in digestion the contents may be too much lacking in acidity to prove hurtful to the germ, and the imperfectly digested morsels enclosing the bacillus may be passed on unchanged into the alkaline intestine, a field especially favorable to the development of tuberculosis. Again, in the intervals between meals, when the acid secretion is arrested, the bacillus in drinking-water may easily pass through the sentinel stomach to develop in the intestines.

8. *Chemical poisons in the tuberculous body.* The soluble chemical poisons in the system are a potent cause.

Lesions and symptoms of tuberculosis. Tuberculosis appears under two great types: the acute and the chronic, the first of which may run a fatal course of four or six weeks, while the second may last for many years. At the outset in the acute form, and for a great length of time in the chronic, the disease process may be confined to one organ or to one region of the body, and, therefore, the symptoms may vary exceedingly according to the particular organ attacked.

In many chronic cases, with the tubercles confined to one organ or locality (lymphatic glands, liver, spleen, pancreas, etc.), the victim may be in good condition, and no signs of disease may be recognized by the owner or veterinarian.

In acute cases, on the other hand, and when the tubercles are generally diffused through the body, there are usually fever, wasting, and emaciation, in addition to the characteristic symptoms of disease in particular organs. The lesions being caused by the colonization and local multiplication of the bacillus, they tend to assume a rounded or nodular form, from which has been derived the name of tubercle. Such nodules may, however, be absent, the diseased product being a diffused infiltration and thickening of the affected part.

The early nodule may vary in size from a millet seed up to a pea or more. It is first red, congested, and firm; soon it may become gray in the centre, though still red outside. With the grayish discoloration comes a gradually extended death of the mass (coagulation necrosis) and disintegration into a more or less soft cheesy looking material (caseation).

In cattle and chickens this cheesy nodule tends to remain firm, and it may even become gritty through impregnation with earthy salts (calcification). Exceptionally it will soften into a semi-liquid, whitish débris resembling an abscess; and this excessive softening is the usual course of tubercle in swine.

In some cases, however, the tubercle does not break down into a dead, cheesy mass, but develops into firm, fibrous, rounded nodules, hanging in clusters from the lungs, inside of the ribs or skin, and known as pearl disease (perl-knoten), grapes, etc. This form is particularly common in cattle. In man may be found nearly all forms of the disease, the primary hard-red congested nodule, the same with its grayish disintegrating centre, the firm, caseated mass, the same further softened into a yellowish, semi-liquid, pus-like mass, and in addition an open, unhealthy sore caused by the breaking down of the tuberculous growth on the skin (lupus), or intestine, etc. Similar tuberculous growths are found on the skin or mucous membrane of the bowels and throat of cattle and other animals. In all the many forms and seats of the disease the bacillus may be found in the affected parts and the morbid discharges from the lungs, skin, open sores, etc.

Symptoms in cattle. 1. *Tuberculosis of the lungs.* In the chronic cases, which are by far the most common, this may last for months and years unperceived, in acute cases it may prove fatal in a month. In recent slight chronic cases there may be no other ground of suspicion than an occasional cough when the animal leaves the hot stable for the cold outer air, when it is suddenly raised in the stall, when it runs for a short distance, when it drinks cold water or eats dusty food. The cough is usually small, dry, wheezing, and may be repeated several times. When run or driven rapidly the animal proves short-winded, yet it may show as good spirits, as clear and full an eye, as smooth a coat, as supple and mellow a skin, as good an appetite, as rich and abundant flow of milk, and as much propensity to fatten as its healthy fellows. An accomplished diagnostician may detect altered sounds on percussion and auscultation of the chest; but from the difficulty introduced by the heavy muscular shoulder, the frequent variations in the size of the heart, the rumbling and crepitating sounds from the stomach and bowels, which, according as they are full or empty, press forward and diminish the size of the lungs and greatly mask or modify the results, even the able practitioner cannot be trusted to detect these, and the case fails to be recognized.

There may be a flow from the nose in which the bacilli should be detected by the microscope, but cattle have a habit of cleaning the nose with the point of the tongue, so that the virulent particles are difficult to secure, and when secured they prove to contain few bacilli, so that a failure to find these would not be so assuring as it would be in man.

As a large proportion of cases of chronic tuberculosis of the lungs are of this kind, the tuberculin test, to be noted below, becomes practically indispensable. When the lungs become more extensively involved, symptoms are more distinct and reliable, and the animal usually falls off in condition, yet in many cases cattle in good condition are killed for beef, and the lungs and ribs are found to be literally covered with clusters of fibrous tubercles (grapes). Usually in advanced cases the hair is dry, lustreless, and erect in patches, especially along the back; the skin is dry, powdery, and rigid, without its customary mellow touch or mobility on parts beneath; the eyes are less prominent and brilliant; the breathing is more or less easily accelerated; the cough, frequent and easily roused, is often gurgling or rattling, and may cause a discharge from the nose of a whitish flocculent; sometimes gritty material, and the flocculi bacilli may be found.

The breath is heavy and mawkish. Pinching of the back at the shoulder or loins may cause wincing, groaning, or cough, as may also pinching above the breastbone or striking of the ribs with the fingers or fists.

Percussion over the ribs reveals spots where there is a lack of resonance, apart from the solid masses of the heart, liver, spleen, and stomach contents, and listening over these spots will detect that variety of morbid sounds familiar to the physician, the most prominent being rubbing, wheezing, creaking, or fine crepitation, mucous rattling, and various blowing sounds. A remarkable feature of tuberculosis, distinguishing it from many other forms of lung consolidations attended by unnatural sounds, is the occurrence of such changes in patches with intervening spaces of sound lung. Ordinary inflammations more commonly attack one portion and spread from that as a centre extending the solidification in one or all directions. Arrived at this stage, the animal usually fails to make flesh satisfactorily on the best feeding, and milk is not only lessened, but becomes poor, blue, and watery.

The tubercles tend also to form more in other organs, notably the lymphatic glands and bowels, and digestion and assimilation being thus seriously interfered with, emaciation advances more rapidly.

This advance may be largely accounted for by the fact that the infecting expectoration brought up with the cough is largely swallowed to affect stomach and bowels. The animal has now a diminished and capricious appetite, irregular, infrequent, slow rumination, and slight bloating after meals. The temperature is frequently higher in advanced stages. Everyone can recognize the consumptive animal in the advanced stage of lung tuberculosis. It is miserable, poor, and wastes day by day; the dry coat of hair stands erect; the harsh, scurfy skin clings tightly to the bones; the pale eyes are sunken in the sockets; tears run down the cheeks; a yellowish, granular, fetid, and often gritty discharge flows from the nose; the breathing is hurried and catching; the breath fetid; the cough is weak, painful, and easily roused. Tapping the ribs with fingers, or first applying the ear, detects far more extensive changes, including in many cases evidences of blowing into empty cavities (vomica) and loud gurgling. Temperature may vary from below normal to 107° F. To give means of diagnosis of tuberculosis from some diseases of the lungs which most resemble it in symptoms (lung worms, hydatids, actinomycosis, etc.) would unduly extend this article without corresponding advantage to my fellow-men. Tuberculosis of the stomach, bowels, and mesentery glands in animals living on milk gives rise to indigestion, fetid diarrheas, bloating, and finally enlargement of the superficial lymphatic glands, and the affection of the lungs if the animal should survive long enough. In older ones there is impaired, irregular appetite and rumination, slight bloating after meals, a tendency to scour when liberally grained, costiveness, alternating with scourings, colics, and usually a more pronounced wasting than in lung diseases. The rectal examination may detect the enlarged mesentric glands.

2. *Tuberculosis of womb and ovaries.* These and their supporting membrane and ligaments are often implicated in the bowel disease, giving rise to undue generative excitement. The affected cow is usually sterile sooner or later, parting with any ovum that may have been impregnated. Her heats may become more intense and last longer—till general tuberculosis sets in.

3. *Tuberculosis of liver, spleen, or pancreas.* The liver is one of the first organs to suffer in infection through the stomach and bowels, and it may be exclusively affected in calves and even in mature animals.

There may be impaired appetite and indigestion, bloating after meals, and in exceptional cases jaundice; but often an indefinite ill health is all that can be detected. Pancreatic and splenic tubercle are marked by similar obscurity of symptoms till the disease becomes generalized.

Tuberculosis of kidneys and bladder may be attended by extra sensitiveness of the loins to pinching and frequent passing of urine, more or less discolored by blood mixed with purulent matter. The microscope may reveal the bacilli a ter they have been stained. Tuberculosis of the throat and pharyngeal glands is a common form in cattle. Attention is drawn by a wheezing breathing, the sounds coming from the throat; the glands around that part are felt to be enlarged unequally on both sides, or shrunken and of gristly hardness, or softened, and even fluctuating on pressure. There is usually a loose gurgling cough and some difficulty in swallowing; small tubercular growths may exist on the lining mucous membrane, and this sometimes extends into the air-passages and involves the larynx. The udder is also liable to disease. A portion of a single quarter is usually first affected, causing a circumscribed swelling harder than the rest of the gland, but not hot or painful; this gradually extends to the whole udder. With this extension the gland becomes harder and the milk lessens, more watery and clotted; the lymphatic glands in front of the udder and behind are enlarged and hardened. It gradually advances till the milk appears watery or grumous, when it is noticed by the milker.

In cattle the lymphatic glands are often found to be tuberculous to the exclusion of the internal organs, and as this form of the disease tends to become chronic it is likely to be overlooked for a length of time.

4. *In addition to those already cited, there may be here named:* The submaxillary glands are almost imperceptible in their natural condition, may swell up to any size, soften, burst, and discharge a cheesy matter. They are most liable to be confounded with actinomycosis of the same region, but do not show the almost microscopic, hard, yellow clusters of the actinomyces. They yield in place the specific bacillus. The glands inside the chest, mediastinal, bronchial, etc., give no easily available symptom. The same may be said of the glands above the sternum and below the spinal column. Of the more superficial ones, the glands in front of the shoulder, above the stifle, external inguinal, the posterior cervical, may be easily noticed if at all enlarged, and may be taken hold of in thin cattle. The deep-seated lymphatics are also liable to disease, but may not be individually referred to; they rarely show as external swellings. Tubercles may appear in any part of the skin. The lymphatic glands may become irritated and congested, and swell, so that their mere swelling should not be sufficient evidence of tuberculosis.

5. *Further evidence is demanded.* The bones and joints are also affected most commonly in young, growing calves. As the disease usually extends from the gristly, growing substance in the bones of the joint surfaces, the trouble is recognized as disease of the joints and cancellated tissues on the ends of the bones. The joints swell and the ends of the bones enlarge and become tender. The disintegrated bone may even crumble and the sharp spicula protrude through the skin.

The above outline of symptoms seen in cattle is rendered necessary by the invariable question: "How can I recognize it?"

THE INTELLIGENT MAN will realize from the partial sketch above that it is no simple matter to diagnose tuberculosis, and not listen to the man that boasts loudly but really knows little.

In the multitude of equivocal and occult cases further tests must be applied, as the microscopic search for the bacillus which has been incidentally referred to, inoculation may be noticed. This consists in the introduction into the peritoneum or other parts of the guinea-pig, or other animal, of some of the suspected product (discharge from the nose, milk, juice from an enlarged gland, etc.). General abdominal tuberculosis should be present in the guinea-pig in thirty days. The tuberculin test gives prompt results and is less open to fallacy.

Tuberculin or Koch's lymph consists in the concentrated sterilized liquids in which the bacillus tuberculosis has been grown. It contains no living bacillus, all germs having been killed by heating; but it does contain the chief poisons which are produced in the tuberculosis body and which bring about all the diseased process in such body. A possible exception may be made of any such poisons as are destroyed by heat if any such there be in tuberculous products. It must be distinctly understood that in every contagious disease there is: 1, the germ which grows and multiplies in the susceptible animal's system, but is not, in itself and by its mere presence, necessarily injurious; and, 2, the products of the life of that germ which may or may not be poisonous. The many germs that enter the body have products that are not appreciably poisonous, and therefore produce no disease, whereas the few that do manufacture poisonous germs cause our different contagious diseases. Tuberculin consists of chemical poisons which the bacillus secretes or manufactures, and on which the force and all the manifestations of tubercle in the tissues are chargeable. Having no living germs, it cannot increase its own substance, nor can it cause tuberculosis in a healthy system, as it is soon thrown out of the body by the kidneys and other channels, and its power for evil is at an end; yet none the less is it the immediate agent through which all the destruction worked by tuberculosis is carried on. Where the bacillus tuberculosis lives and multiplies in the animal body these chemical poisons are being constantly formed, and thus its pernicious action is continuous, not only in the seat of the tubercle, but through the whole system.

The tuberculin test is based on the fact shown by Koch that it increases the activity of the disease process in tubercle and affects the whole animal body, producing a reaction or rise of temperature in a marked degree. On the ordinary tubercle as seen on the surface, the frequent use of tuberculin produced a more active process of cell growth, leading to degeneration and death, so that there was more speedy transition from red congested nodule, through the grayish degeneration into the dead, cheesy mass, cut off from all blood circulation. But deeper tubercles usually exist, and these are similarly stimulated and their degeneration hastened by the tuberculin, thus rendering tuberculin almost useless as a curative agent, but makes it of the highest value as a test of tuberculosis in animals. The minute dose which has no effect on a healthy cow, horse, or pig, when employed on the slightly tuberculous one produces an acceleration of the disease process and in eight to fifteen hours a material rise of temperature. This has been now employed on thousands of cows, and those that use it most value it most highly. I have used it on a large number of cattle during the past year, and while examining a herd of domestic cows by the ordinary means, that is, without the aid of the tuberculin test, discovered one cow that I suspected, and ordered her to be tested with tuberculin. Through a mistake of the herdsman, that was not discovered till after the examination by the tuberculin test, he submitted the wrong animal; she was a cow of six years old, apparently in good health, and would dress about 700 pounds of beef, and did not show the slightest trace of tuberculosis by ordinary means, and when tested by Koch's lymph she proved to be diseased, her temperature rising to 108° F. fifteen hours after the injection. The *post mortem* showed tubercles in most of the lymphatic glands, lungs, kidneys, adipose tissue, and, lastly, the liver; it contained a tubercle which I have preserved in my office, weighing several pounds.

This led to a test of the rest of the herd with tuberculin, and I discovered that 50 per cent. were affected. This is only one case out of a large number which, if I was to relate, would occupy too much space. In a herd of 37, reaction after tuberculin occurred in 31, while 6 gave no reaction. When killed the 6 proved sound, and the 31, without exception, tuberculous.

Infection by bacilli in meat and milk. First, however, it will be instructive to compare the geographic distribution of cattle and that of tuberculosis, and show the intimate relations of cattle to man as a potent agent in the extension and maintenance of consumption in the human family. To the student of this subject it is plain that where cattle are few or absent, consumption is relatively less prevalent in man. In northern Norway, Sweden, Lapland, and Finland, where reindeer constitute the chief farm stock, about Hudson Bay and in the islands of the Pacific, where no cattle exist, and in the Scottish

Hebrides, Iceland, and Newfoundland, where cattle are few, tuberculosis is less prevalent in man.

In Algiers (a resort for consumptives) the cattle are few and live in the open air, apart from the cities, and tuberculosis does not increase among the natives. In Italy (another resort for consumptives), where cattle are housed, tuberculosis has become the scourge of man and beast. In Australia (a great resort for English consumptives) the disease, formerly unknown, has become exceedingly prevalent, and the same is becoming true of our own Minnesota, formerly so lauded as favorable to weak lungs.

Our Northwest Indians furnish the most striking illustration of infection derived from cattle and fostered in man by unhygienic surroundings.

Dr. Treon, in the *American Practitioner*, describes the poor, emaciated, diseased animals furnished to the tribes, how the Indians eat the liver, tallow, and entrails raw and fresh, and how the carcass is dried, pounded, and packed in the skins, to be eaten later without cooking. The meat is eaten though the animal may have died of disease. Dr. Holder, in the *Medical Record* (August 13, 1892), gives the Indian mortality from consumption as 50 per cent. of all deaths at Green Bay, Wis., Tulalip, Wash., and Western Shoshone, Nev. He says that at Lower Bruti, Dak., scrofula is present in 60 per cent. of the Sioux under twenty-one years, and that at Crow Creek, Dak., 50 out of a total Indian population of 1200 die yearly of consumption and scrofula. These are extreme examples, it is true, in which the transmission and fostering of the disease by cattle are extended and aggravated by overcrowdingand every imaginable unhygienic condition among the human consumers.

LOCKPORT, N. Y., December 12, 1894.

